# Selections and Abstracts

**Published:** 1897-08

**Authors:** 


### SELECTIONS AND ABSTRACTS.

### OPHTHALMOLOGY AND OTOLOGY.

EDITED BY ALEX. W. STIRLING, M.D.

A CASE OF PYEMIA AFTER ACUTE SUPPURATION OF THE EAR. OPERATION: RECOVERY.—(Röpke, *Archives of Otology*, Vol. XXV., No. 4.)

In this case, the patient, a female, aged 26, contracted a severe head cold, followed the next day by violent pain in the left ear, and by profuse discharge. Ten days after the onset of the trouble she consulted the author, complaining of sleeplessness, loss of appetite, lassitude, throbbing in the left ear, and pain in the left temple and back of head. Upon examination, the left membrane was found markedly swollen in its posterior segment, and perforated in its anterior. The mastoid process was not swollen, but apical pain was complained of. Temperature, 37.6 degrees C.; pulse, 100.

The patient, although under careful treatment, gradually became worse, and was eventually admitted to hospital.

The mastoid and the region immediately behind it was not swollen. The neck was freely movable, no swelling in any of the cervical glands was found, nor could any cord in the jugular be felt. Pain was, however, complained of at the apex of the mastoid process. The usual mastoid incision was made, and the flaps well retracted. A small quantity of pus oozed from the dilated emissary mastoid vein. The process was opened, and thick pus and granulations immediately found in a large cavity extending backward to the region of the lateral sinus, and below to the mastoid apex. The wall of the sinus was punctured, and found to contain a solid thrombus. An uninterrupted recovery followed.

In his remarks upon this case the author says that the chill, the sudden rise of temperature, and then the equally rapid fall to the normal, the rapid pulse, the enlarged spleen, and the dry, cracked, anl thickly coated tongue, all supported the diagnosis of otitic pyemia. Although the sinus was found thrombosed, the author inclines to the idea that the origin of the pyemia was independent of this thrombosis, but was propagated from the veins of the bone. The subsequent favorable course of the disease also corroborates the view that the pyemia occurred from an osteophlebitis, for, after clearing out of the primary seat of the disease, only one chill occurred.—*Medical Chronicle.*

---

### OCULAR DISEASE FROM GOUT.

Wagenmann, A. Jena. *(Arch. of Ophthal.*, Jan., 1897.) It has but lately been realized that true gout may cause ocular disease. Instances are more frequent in England and France.

The gouty diathesis, whether the symptoms be typical or atypical, must be demonstrable in order to make the diagnosis. The familiar involvement of the metatarso-phalangeal joint of the great toe, changes in other joints, disturbances of the digestive tract, muscles, organs of respiration, circulatory or nervous system, or gouty cachexia may be present. Neuralgia, lumbago, spasm of the calves, early atheroma of the vessels, migrain, eczema, etc., may occur and are of diagnostic value. Urinalysis reveals free uric acid and excess of urates. The tissues of the eye which are most liable to be involved are the choroid and sclera, but the following may also occur: Lithiasis and eczema of the lids, keratitis senilis as a form of peripheral corneal opacity or a circulus seniles, scleritis, iritis distinguished by a great tendency to hemorrhage and excessive painfulness, choroiditis, cyclitis, retinitis, retino-choroiditis, cataract, particularly presenile glaucoma, thrombosis of the retinal vessels, migrain with visual disturbances and paralysis of the ocular muscles. The decidedly favorable effect upon glaucoma of antiarthritic treatment in these cases is striking. Often the glaucomatous process increases despite eserin and accurate correction of refraction for distance and near, until an inflammatory outbreak transpires and the symptoms subside only when general treatment of the gouty diathesis is resorted to systematically. Gout may predispose to glaucoma by causing certain vascular changes, producing a tendency to inflammation in the arterial tunics and endothelium and by inducing rigidity of the sclera.—*H. V. W. in Annals of Ophthalmology, April, 1897.*

---

### HYSTERIA WITH STRABISMUS AND PTOSIS.

E. Hitzig, of Halle, points out that disorders of the oculomotor mechanism are rare in hysteria, though diminution of the visual field, and other phenomena due to fatigue of the retina, are very common. The patient, a Polish laborer, aged 36, had previously suffered from inflammation in the eyes." He had indulged rather freely in spirits. In August, 1895, he wounded himself in the leg. The wound healed rapidly, but unfortunately he got the idea that, as there had been no suppuration, inflammation would occur elsewhere. After a week, in reality, he got inflammation in the eyes, followed by diplopia and ptosis. This condition lasted a long time without distressing him, but when he found himself unable to see two of his children, who had returned home in September, 1896, he attempted suicide by drowning, and for two days appeared insane. On admission (September, 1896) there was complete ptosis on both sides; both eyes were turned inwards and downwards. Later, in November, the pupils did not react to light, and the patient professed to see nothing. There was no evidence of any spasm of the orbicularis muscles. There was partial unilateral loss of taste and smell. Tactile sensation was absent over the whole of the body, and there was analgesia in the right arm, left leg, and left half of the trunk, neck and head. Temporary improvement in the eye symptoms had followed chloroform narcosis in October, but their complete disappearance followed electricity and suggestion in November. A temporary partial hysterical deafness was likewise recovered from, and in January, 1897, the patient's condition was practically normal in every respect. Some of the eye symptoms were evidently due to spasm, namely, the squinting and the myosis. Charcot in 1888 still believed that all so-called hysterical unilateral palsies of the facial, lingual, and oculomotor muscles were in reality hemispasms. Charcot and his followers had, however, afterwards to modify their views, and in the present case Hitzig attributes the ptosis to hysterical paralysis of the levator muscles of the eyelids.—*British Med. Journal.*

### ACUTE GLAUCOMA ASSOCIATED WITH LARGE NASAL POLYPUS.

Patient, Mr. M., aged 55, in fairly good health, applied for advice in relation to the vision of his right eye, which had recently become quite dim. There had been attacks of dimness recently but they had subsided, but as these attacks had become more frequent he became alarmed. He had always enjoyed excellent vision and was wearing the ordinary convex glasses suited to his age. Vision in the right eye was 0.2. The pupil was moderately dilated but responded sluggishly to light. Tension was + 1 ?; tension of the left eye was normal. The ophthalmoscope revealed no cupping. The nasal twang about his voice induced me to examine his nose, and I found in the right side a very large and solid mucous polypus which quite filled its lumen. It was quite impossible for him to breathe through this side. He said this state of affairs had existed for a long time, but as he could breathe through the other side he had paid little or no attention to it.

Eserine was ordered for the right eye and the next day, February 24, 1891, vision was reduced to 0.1. Tension same as yesterday. February 26, eserine has been used for the past two days and V. = 0.3. There is no turbidity of the media. Removal of the nasal polypus was urged on the grounds that it was necessary for healthy respiration and that there might be some indirect connection between the glaucoma and the pressure in the nasal cavity. This was done at once and the next day the eye was decidedly improved. Vision was 0.7, and tension was normal. February 28, V. = 0.8. The glaucomatous symptoms seemed to disappear as soon as the polypus was removed and his eye promptly regained normal vision.

Two or three years later he was a client of mine for conjunctivitis, but there was no return of the increased tension or of the spells of dimness which he had prior to the operation. Was this a reflex irritation?—*S. C. Ayres, in Am. Jour. of Ophth.*

---

### OSTEOMATA OF THE AUDITORY CANAL AND THEIR TREATMENT.

Dr. E. J. Moure states that the osseous obstructions of the auditory canal are usually formed of impacted tissue of ivory consistency, and are difficult to remove with cutting instruments. (*Revue*

*Hebd. de Laryng. d'Otol. et de Rhin.*, Sept. 26, 1896.) It is better to remove them with a gouge than to try to reduce them with the drill, or by the numerous methods generally recommended by authors.

The radical and complete eradication is as important here as in the osseous tumors of the nasal fossa and of the face. The author relates two cases in reference to men 45 and 50 years old, who had osteoma of the canal, and at the same time suppurative otitis media. The skin was incised and a gouge passed through this opening and pushed in until the tumor was loosened and detached. If the osteoma is very near to the tympanum, it is more prudent to free the pavilion and the cartilaginous canal before attempting the operation of the tumor.—*Laryngoscope.*

### ENUCLEATION AND EXENTERATION.

Pfluger, E. (*Wein. Klin. Woch.*, Feb., 1897.) In order to prevent sympathetic ophthalmia, enucleation is the only proper operation. Many cases have resulted fatally from purulent meningitis despite antisepsis and enucleation. The advantages of exenteration, a large and mobile stump and less deformity, are not sufficient to counterbalance the increased danger in these cases. Friedrich Stocker concludes from 41 cases with enucleation, that meningitis sometimes occurs notwithstanding enucleation, but not as a result of it. The danger of spreading infection by breaking through the line of demarcation is obviated by careful antisepsis and drainage.—*H. V. W. in Jour. of Ophthalmology, April.*

### MISCELLANEOUS.

### HOW LONG IS SYPHILIS CONTAGIOUS?

I may state, without fear of successful contradiction, that there are no well authenticated cases on record of syphilis occurring in the second generation as a result of heredity. I again state that there are no cases on record where vaccino-syphilis has been transmitted from children over eight months of age. Hence, no matter what conditions may confront us in acquired syphilis, it is almost certain that in hereditary syphilis its power of transmission is lost by the end of the first year of infection.

The question as to acquired syphilis is one which is far more perplexing in its entirety. The majority of authors with whose teachings we are familiar hold that from three to five years are required before the contagiousness of syphilis is at an end, but state that in exceptional cases it has been found to remain over a period ranging from six to twenty years; such cases undoubtedly occur, but they must be rare indeed.

Mr. Jonathan Hutchinson, before the third International Congress of Dermatology, London, made bold the statement that syphilis had generally lost its contagiousness at the end of the first year of infection; he further stated that he always allowed his patients to marry at the end of the second year, and in but two instances had the children shown any signs of syphilis as a result of such marriages. I do not desire to question the statement of so great and good a man as Mr. Hutchinson, yet at the same time I beg leave to criticize and state that such advice is not without danger, and could hardly be called conservative.

I now call to mind four different cases wherein syphilis had been disseminated at two, three, five and one-half, and seven years, respectively, after the initial lesion had occurred. I would state that in the case where syphilis was contracted five and one-half years after the initial lesion, three men were the victims. When the woman, the fourth victim and disseminator of the disease, came under my observation, there was situated just off to the left of the meatus urinarius an ulcer the size of a silver dime. She also was the unfortunate possessor of numerous scars, pretty well scattered over her entire body, in sizes ranging from that of a spilt pea to that of a silver dollar and larger, some of them being slightly discolored, but the majority pale and shiny in their appearance. She assured me that her disease had been terrible in the extreme, and that the scars I saw had been there for a period of three years, and from their appearance I do not doubt her word. I know that in this instance it may be charged that this was a case of reinfection; but these cases are so rare, and besides her general health being apparently good, she was treated locally (and, for reasons which I need not state, was given no constitutional treatment whatever), and the lesion healed in about two weeks. I have observed her for about four years since that time, and she has shown no manifestations of syphilitic disease.

The so-called gumma or tertiary period is usually considered to carry no infection; but, from what I have just related, I hardly think that I would care to make the experiment upon my own person. I will further state that the first, second and fourth cases mentioned were all of hereditary syphilis transmitted through marriage.

The case of infection at the seventh year is peculiarly an interesting one. The father had acquired syphilis seven years prior to marriage with a perfectly healthy young woman; as a result of this marriage a child was born one year afterward, manifesting all the symptoms of hereditary syphilis. At the age of three years this child became hemiplegic, from which she has never recovered. The second child two years later was born healthy and has remained so; two abortions occurred after the second child was born; but at the end of three years another child was born, with a distinct syphilitic rash, snuffles, mucous patches, etc. Six weeks after the birth of this last child the mother, heretofore having remained perfectly well apparently all these years, developed a most beautiful macular eruption all over the body, with enlarged glands, sore throat, etc.; which case seemingly proves an exception to the well-known Colles law.

In conclusion I would like to assert dogmatically that treatment does remove not only the manifestation of this disease, but the disease itself, and with it its contagiousness, some eminent authorities to the contrary. I believe that syphilis loses its virulency as time progresses, and that by the end of the sixth year it has practically lost its contagiousness, with here and there a single exception. I further believe that where patients have been well treated, they may marry, all things being equal, in three or four years after the primary lesion has occurred.—*Dr. C. T. Drennen, Memphis Med. Monthly.*

---

### THE TECHNIQUE OF VAGINAL SECTION, IRRESPECTIVE OF HYSTERECTOMY, FOR DISEASED APPENDAGES AND SMALL PELVIC TUMORS.

This is the subject of a paper presented at the recent meeting of the American Medical Association, Section on Gynecology, by Dr. Augustin H. Goelet, of New York. He says anything that will lessen the shock in operations, reduce the mortality, and shorten convalescence is a decided advance in our methods, which is true of vaginal section when done under proper indications. But it is an error to think that vaginal section will supersede abdominal section. Both have their natural limitations based upon,

1. The ease with which the operation may be completed.

2. The complications which may develop as the operation proceeds.

3. The danger which the operation incurs.

4. The immediate as well as the remote results including the necessity for drainage.

The advantages of vaginal section are stated as follows:

1. The patient will more readily consent to a vaginal section, hence hopelessly diseased organs may be removed earlier and the mortality is thereby lessened and her suffering relieved earlier.

2. The surgeon would not hesitate to advise vaginal section in conditions which do not seem to present sufficient gravity to warrant the risk of abdominal section.

3. Where exploration of the pelvis is necessary to clear up the diagnosis where the diseased condition is not extensive and where the adhesions are not dense or numerous.

4. When subsequent drainage is certain to be required it is more perfectly secured through a vaginal incision.

The operation is applicable in the following conditions:

1. For small pelvic tumors which are or seem to be solid in part or entirely so.

2. In ovarian cysts of considerable size, since the fluid can be evacuated and the sac withdrawn through a small vaginal incision.

3. For pyosalpinx, hydrosalpinx and hematosalpinx.

4. For cystic or otherwise enlarged and diseased ovaries.

5. For the removal of small subperitoneal fibroids by myomectomy.

6. For drainage of pus accumulations situated low down in the pelvis.

7. For hematoma and hematocele, including ectopic gestation.

8. For pelvic exudations which resist other means for their removal. He has had excellent results from persistent faradization in these last named conditions, hence he believes that surgical interference is seldom required where suppuration has not occurred.

The technique of the operation is described in detail and very concisely.

In conclusion the author states that complete union of the vaginal incision usually takes place in from six to eight days, even when the gauze drain is employed, and the patient may be gotten out of bed at the end of two weeks. It is, he says, the exception when any rise of temperature follows this form of vaginal section, and convalescence is, as a rule, rapid and uneventful.

---

### HAND DISINFECTION.

Prof. R. F. Weir *(Medical Record*, April 3, 1897) describes the following method of hand disinfection, which has well stood the test of time clinically, and has proved itself by repeated culture tests to be among the most reliable of agents for this purpose:

After a thorough scrubbing with hot running water, green soap and a brush, aiding the cleansing under and about the nails with a pointed brush, like the end of rattan or soft wood, about a scant tablespoonful—*i.e.*, a large pinch of the ordinary commercial chloride of lime or bleaching powder is put in the palm of the hand, and on it is placed a crystal of the carbonate of sodium (common washing soda) about one inch wide and one-half inch thick, or of a size that will permit it to be easily rubbed about like soap. A little water is now added, to make a thick cream, and this is rubbed up and down the palms and hands and arms, until the little rough grains of the chloride of lime disappear or markedly diminish, or until the creamy fluid thickens into a pasty layer. Another ready way to determine when to cease using the soda, is to cast it aside when a sense of coolness is felt in the palms, for when the cream is first made a sensation of heat is noticed. The procedure occupies from three to five minutes. Before washing the paste off in sterile water, I prefer to use a sterilized rattan cleaner to rub it under and around the nails. When finally cleansed in the sterile water, the hands will be found smooth and soft and smelling of chlorine. If the odor persists after the operation, neutralization can be had, or before this if any reason exists, by a (sterile) weak one-fifth per cent. solution of aqua ammonia. There is seldom any irritation of the skin wollowing this chlorine asepsis. Should such occur, bathing the parts (after the operation) with water and anointing with non-petrolic oil will at once remove it. Depending somewhat on the sensitiveness or thickness of the skin, the procedure can be employed, if required, three or four times a day, without marked irritation.—*Internat. Journal of Surgery.*

---

### THE TREATMENT OF FRACTURES.

Dr. W. L. Estes, in an article on the treatment of fractures, in the *International Journal of Surgery*, says:

1. Unless a fragment is threatening to break through the skin, the fracture should never be reduced except by a physician, and then only when apparatus is at hand to keep the parts in permanent apposition.

2. Men carrying an injured person should not keep step, as the jar to the wounded part is much greater.

3. Strychnia for shock, morphia for pain, but *no alcohol.*

4. Always give anesthetics for reduction of a simple fracture. It is better and easier to reduce and set compound fractures without anesthesia.

5. It is very rarely necessary to make a patient go through the double agony of "temporary" and "permanent" setting of the broken bones.

6. In simple fracture gentle rubbing of the ends will assist in getting rid of shreds of tissue which invariably are caught there.

7. Nowadays a surgeon will rarely be satisfied that a bone is properly set, until verified by the X-rays.

8. Plastic splints, preferably plaster of Paris, are surely the best apparatus when they can be applied.

9. Ambulant treatment is coming more into vogue. No simple fractures require constant confinement to bed, except of the innominata and upper third of the femur.

10. It is not necessary and sometimes very harmful to "wait for swelling to disappear" before putting on a permanent dressing.

11. A well applied splint with good apposition of fragments should not be removed too early. It is not necessary to apply massage early in ordinary cases.

12. Proper time for massage is two or three weeks after fracture of upper extremities and four or five weeks for lower extremities—if the bones are slow to unite firmly.—*Maryland Medical Journal.*

---

### KRYOFIN.

The latest antipyretic is *Kryofin.* Chemically, this drug is closely allied to phenacetin. It occurs as white, odorless, tasteless crystals, soluble in 52 parts of boiling and 600 parts of cold water. The solution has a sharp, bitter taste. The usual dose is 7.5 grains and the antipyretic effects of this amount are greater than those obtained by 15 grains of phenacetin. No unpleasant symptoms have been observed to follow its use. In addition to its antipyretic action, kryofin has been found to have an analgesic power, and is used with benefit in sciatica and in alcoholic neuritis. In acute and chronic articular rheumatism it failed to relieve the pains.—*Medical News.*

---

Professor Koch, the bacteriologist, has just made his report giving the result of his investigations into the bubonic plague which has been ravishing India. He says that the bacilli possess but little vitality outside of the bodies of men and animals, and that Professor Haffkine's serum possesses undoubted protective qualities. Professor Koch states that his report is founded on the results of experiments on fourteen hundred cases.—*Scientific American.*

